# Awareness and Hygiene Practices Among Contact Lens Wearers: A Population-Based Cross-Sectional Survey

**DOI:** 10.7759/cureus.54723

**Published:** 2024-02-22

**Authors:** Osama Albasheer, Ibrahim M Gosadi, Ismail Abuallut, Fouad F Khawaji, Abdullah J Almalki, Alhareth K Muqanna, Abdulrahman A Otaif, Mousa M Abdali, Abdulmalik M Almukhashi, Khaled M Refaie

**Affiliations:** 1 Department of Family and Community Medicine, Jazan University, Jazan, SAU; 2 Department of Surgery, Jazan University, Jazan, SAU

**Keywords:** eye dryness, eye redness, practice, knowledge, contact lens

## Abstract

Background

More than 150 million individuals worldwide wear contact lenses (CL) for therapeutic as well as cosmetic purposes. Researchers have linked failure to adhere to CL care instructions with outbreaks of serious eye infections. In the current study, we assess the consequences of inadequate cleanliness as well as awareness of and adherence to appropriate CL hygiene.

Methods

This is a cross-sectional descriptive study conducted in the Jazan region, southwest Saudi Arabia, during the period between October 2022 and April 2023.

Results

Out of a total of 718 participants, 391 (54.4%) were wearing CL. Of these, 318 (81.33%) CL wearers were female, while 73 (18.67%) were male. Among the CL wearers, 196 (50%) were aged 24 and younger. The overall knowledge was higher in females, with 320 (74.6%) having a high level of knowledge, compared to 195 (67.5%) males (p-value = 0.044). The level of knowledge was higher in those who had had an eye examination before using CL. Regarding practice, 202 (63.5%) females demonstrated better practices, while only 32 (43.8%) males did (p-value = 0.002). Variations in the adequacy of practice regarding CLs were identified according to the duration of CL use and the risk of suffering from eye dryness (p-values <0.05).

Conclusions

The participants’ overall knowledge and practices were good. This should be encouraged by performing an eye examination before wearing CL and demonstrating good wearing habits in terms of hygiene behavior.

## Introduction

Contact lenses (CLs) are an ocular prosthesis worn by more than 150 million individuals globally [1]. They can be used for medical reasons, such as vision correction, or for cosmetic purposes, such as changing the eye color [2]. CLs help people improve their vision without affecting their appearance or interfering with various sports and activities. Specially designed CLs enhance the ability to focus and may be useful in controlling myopia in children [3].

The abundance of options available, not only in terms of lens type and material but also in terms of availability at more locations across the nation and at prices far lower than in the past, are likely factors in the popularity of CL wear [4]. Because the cornea is a sensitive area to which the CLs are applied directly, proper application and hygiene regarding their use are important to avoid complications.

Some studies show that good practices when using CLs include cleaning hands before wearing and after removal of CLs, not sharing CLs, not sleeping while wearing CLs, and cleaning CLs with preserving liquid [5-9]. Other studies show that poor practice when using CLs can lead to complications such as microbial keratitis, eye redness, itching, dryness, corneal inflammation, and visual loss [10-12].

Because researchers have linked failure to adhere to CL care instructions to outbreaks of serious eye infections, there is a need to assess public awareness and hygienic behavior to ensure healthy eyes while wearing CLs.

To the best of our knowledge, researchers have yet to extensively evaluate public awareness and practice among adult Saudi CL users. The current study aims to assess the level of knowledge about contact lens use among the adult population in Jazan, Saudi Arabia, and to identify characteristics of participants associated with the level of knowledge. Additionally, this study aims to assess the adequacy of contact lens-wearing practices, the consequences of inadequate cleanliness, and the characteristics of participants according to the adequacy level of practice.

## Materials and methods

Study design and settings

This cross-sectional descriptive study was conducted in the Jazan region, southwest Saudi Arabia, from October 2022 to April 2023.

Study population

The study population comprised a total of 718 participants.

Inclusion criteria

Adults aged ≥ 18 years living in the Jazan region of Saudi Arabia, both male and female, Saudi and non-Saudi, willing to participate in the study, were included regardless of their use of CLs.

Exclusion criteria

Children under 18 years, adults with poor communication skills, those with serious physical or mental health problems, or those unwilling to participate were excluded.

Sample size

The sample size was calculated using Raosoft software, with the following assumptions: an estimated population size of individuals aged ≥ 18 years of 20,000, a 95% confidence level, a margin of error of ±5%, and an estimated response rate of 50%. The minimum sample size was set at 377, multiplied by two to ensure an adequate sample.

Data collection process

Data were collected through a self-administered online questionnaire disseminated via social media platforms (WhatsApp, Telegram, X, and others), after obtaining informed consent from participants. The data were recorded in Microsoft Excel and stored anonymously, as the questionnaire did not include any personal information such as names, ID numbers, or any specific information that could lead to participant identification.

Data collection tool and study variables

A validated questionnaire, which demonstrated adequate reliability and validity in a previous study, was used. Permission was obtained from the corresponding author via email. The questionnaire included 45 questions divided into four parts: sociodemographic characteristics, knowledge, practice, and complications. Those wearing CLs answered all four parts, while those not wearing CLs answered only the first two. The first part included questions on sociodemographic characteristics such as age, sex, and monthly income. The second part assessed knowledge on topics such as the necessity of an eye examination before using CLs, interval for periodic eye examinations after CL use initiation, and knowledge about CL use and expiry, including preserving liquid use and expiry. Additionally, it covered the method of wearing CLs, safety, and the risk of infection when wearing CLs while sleeping, showering, or swimming. Knowledge scores were categorized as high (71%-100%), intermediate (51%-70%), or poor (0%-50%) based on participants' answers. The third part evaluated CL usage practices, including habits of sharing CLs, using CLs past their expiration date, and hand hygiene before wearing or removing CLs. Practice scores ranged from zero to nine, with a mean of 4.9; scores of 4.9 and above indicated better practice, while scores below 4.9 indicated poorer practice. The fourth part inquired about complications such as eye dryness and redness.

Statistical analysis

Data were manually verified, coded, reviewed, and entered into a computerized database for analysis using IBM SPSS Statistics (version 25). Descriptive statistics were calculated for study variables, including frequency and percentage for qualitative variables and mean and standard deviation for quantitative variables. The chi-square test of significance was applied as appropriate, with a p-value of <0.05 denoted as statistically significant.

## Results

Demographic characteristics of participants

Table 1 represents the demographic characteristics of participants: 718 individuals agreed to participate in the current study. Of the total, 391 were wearing CLs, of whom 318 were female while 73 were male. Of the CL wearers, 196 were aged 24 and younger, 388 were Saudis and 209 were from the urban region, 274 were university students, and 202 had a monthly income of less than 5000 SAR.

**Table 1:** Socio-demographic characteristics of study participants (n=718), Jazan, Saudi Arabia. The data has been represented as Number (N) and Percentage (%). P-value <0.05 is considered significant. (SAR)*= Saudi Arabian Riyal.

Variable		Do you use contact lenses?	
		Wearers N(%)	Non Wearers N(%)
Gender	Male	73 (25.3%)	216 (74.7%)
	Female	318 (74.1%)	111 (25.9%)
Age	24 or younger	196 (51.9%)	182 (48.1%)
	Older than 24	195 (57.4%)	145 (42.6%)
Nationality	Saudi	388 (54.6%)	323 (45.4%)
	Non-Saudi	3 (42.9%)	4 (57.1%)
Place of living	Village	182 (48.1%)	196 (51.9%)
	City	209 (61.5%)	131 (38.5%)
Educational level	Elementary school	2 (40%)	3 (60%)
	Middle school	3 (50%)	3 (50%)
	High school	85 (51.5%)	80 (48.5%)
	University	274 (54.3%)	231 (45.7%)
	Postgraduate	27 (73%)	10 (27%)
Monthly income (SAR)*	Less than 5000	202 (52.6%)	182 (47.4%)
	5000-10000	100 (65.8%)	52 (34.2%)
	10000-15000	54 (52.9%)	48 (47.1%)
	More than 15000	35 (43.8%)	45 (56.3%)

CL-wearing knowledge and related factors

We observed significant variation in the level of knowledge with regard to gender difference (p-value = 0.044). In females, the level of knowledge was excellent in 320 (74.6%), average in 51 (11.9%), and poor in 58 (13.5%), whereas in males, the level of knowledge was excellent in 195 (67.5%), average in 35 (12.1%), and poor in 59 (20.4%). There were no significant differences with regard to age, nationality, residency, education level, and monthly income (p-values: 0.761, 0.589, 0.218, 0.062, and 0.693, respectively) (Table 2).

**Table 2:** Determinants of contact lens knowledge among study participants (n=718), Jazan, Saudi Arabia. The data has been represented as Number (N) and Percentage (%). P-value <0.05 is considered significant. (SAR)* = Saudi Arabian Riyal.

Variable	Knowledge (N/P)			P-value
	Poor	Intermediate	High	
Gender				
Male	59 (20.4%)	35 (12.1%)	195 (67.5%)	0.044
Female	58 (13.5%)	51 (11.9%)	320 (74.6%)	
Age				
24 or younger	63 (16.7%)	48 (12.7%)	267 (70.6%)	0.761
Older than 24	54 (15.9%)	38 (11.2%)	248 (72.9%)	
Nationality				
Saudi	116 (16.3%)	86 (12.1%)	509 (71.6%)	0.589
Non-Saudi	1 (14.3%)	0 (0%)	6 (85.7%)	
Place of living				
Village	53 (14%)	47 (12.4%)	278 (73.5%)	0.218
City	64 (18.8%)	39 (11.5%)	237 (69.7%)	
Educational level				
Elementary school	2 (40%)	1 (20%)	2 (40%)	0.062
Middle school	3 (50%)	1 (16.7%)	2 (33.3%)	
High school	33 (20%)	14 (8.5%)	118 (71.5%)	
University	73 (14.5%)	68 (13.5%)	364 (72.1%)	
Postgraduate	6 (16.2%)	2 (5.4%)	29 (78.4%)	
Monthly income (SAR)				
Less than 5000	62 (16.1%)	43 (11.2%)	279 (72.7%)	0.693
5000-10000	22 (14.5%)	18 (11.8%)	112 (73.7%)	
10000-15000	22 (21.6%)	14 (13.7%)	66 (64.7%)	
More than 15000	11 (13.8%)	11 (13.8%)	58 (72.5%)	

The participants reflected different information with regard to CL wearing (Table 3); out of 391 CL wearers, the number of participants who used CLs for medical uses was 93 (23.8%), cosmetic 198 (50.6%), and medical + cosmetic 100 (25.6%). Almost three-quarters, 287 (73.4%), of the wearers agreed on the importance of having an eye examination before CL wearing, and 147 (37.5%) suggested having a periodic checkup every six months. The internet 92 (23.5%), friends 86 (22.06%), and families 83 (21.2%) represented the main source of information.

**Table 3:** Information related to contact lens wearing (n=391). The data has been represented as Number (N) and Percentage (%).

Variable	Response	N	P-value
Indication for contact lens use	Medical	93	23.80%
	Cosmetic	198	50.60%
	Medical + Cosmetic	100	25.60%
Having an eye examination before CL wearing	Yes	287	73.40%
	No	104	26.60%
Periodic check up	Yes	147	37.60%
	No	244	62.40%
Source of information	Internet	92	23.50%
	Doctors	51	13.04%
	Family	83	21.20%
	Friends	86	22.06%
	Others	79	20.20%

CL practices and related factors among the study participants

Table 4 presents the CL practice among the participants; regarding sharing CLs with others, 284 (72.7%) of the wearers never or rarely shared CLs with others, whereas 63 (16.1%) always or usually shared CLs with others. Over three-quarters, 319 (81.6%) cleaned the CLs after each use, and only 20 (5.1%) rarely or never cleaned them after each use. The majority of the wearers, 335 (85.7%) washed their hands before wearing or removing CLs, whereas only 14 (1.8%) never washed their hands before wearing or removing them. Regarding the use of CL preserving liquid for cleaning, 351 (89.8%) used it and only 18 (1.5%) never used it.

**Table 4:** Contact lens-related practice of the study participants (n=391), Jazan, Saudi Arabia The data has been represented as Number (N) and Percentage (%).

Variable	N (%)				
	Always	Usually	Sometimes	Rarely	Never
Do you replace contact lenses after their expiry date?	220 (56.3%)	77 (19.7%)	48 (12.3%)	21 (5.4%)	25 (6.4%)
Do you share your contact lenses with your family and friends?	26 (6.6%)	37 (9.5%)	44 (11.3%)	64 (16.4%)	220 (56.3%)
Do you clean your contact lenses after each use?	217 (55.5%)	102 (26.1%)	52 (13.3%)	11 (2.8%)	9 (2.3%)
Do you use the solution for contact lenses when cleaning them?	292 (74.7%)	59 (15.1%)	22 (5.6%)	12 (3.1%)	6 (1.5%)
Do you replace the lens-preserving liquid after its expiration date?	221 (56.5%)	73 (18.7%)	44 (11.3%)	21 (5.4%)	32 (8.2%)
Do you clean your hands before wearing or removing contact lenses?	271 (69.3%)	64 (16.4%)	42 (10.7%)	7 (1.8%)	7 (1.8%)
Do you clean your contact lens case?	197 (50.4%)	92 (23.5%)	74 (18.9%)	21 (5.4%)	7 (1.8%)
Do you move your contact lenses with your fingers before soaking them in the preserving liquid?	110 (28.1%)	66 (16.9%)	58 (14.8%)	62 (15.9%)	95 (24.3%)
Do you replace the preserving liquid when storing your contact lenses after each use?	184 (47.1%)	82 (21%)	71 (18.2%)	38 (9.7%)	16 (4.1%)

Gender and duration of CL use during the day are significantly related to the CL practice (p-value = 0.002 and 0.003, respectively). We present other factors in Table 5.

**Table 5:** Determinants of contact lens practice among the study participants (n=391), Jazan, Saudi Arabia The data has been represented as Number (N) and Percentage (%). A p-value <0.05 is considered significant.

Variable	Practice N (%)		P-value
	Worse	Better	
Gender			
Male	41 (56.2%)	32 (43.8%)	0.002
Female	116 (36.5%)	202 (63.5%)	
Social status			
Single	91 (39.9%)	137 (60.1%)	0.071
Married	53 (36.8%)	91 (63.2%)	
Widowed	5 (71.4%)	2 (28.6%)	
Divorced	8 (66.7%)	4 (33.3%)	
Do you use contact lenses under the advice of an ophthalmologist?			
Yes	43 (34.7%)	81 (65.3%)	0.132
No	114 (42.7%)	153 (57.3%)	
What kind of contact lenses do you use?			
Daily	37 (35.9%)	66 (64.1%)	0.107
Weekly	22 (55%)	18 (45%)	
Monthly	98 (39.5%)	150 (60.5%)	
When do you use contact lenses?			
Daily	29 (40.8%)	42 (59.2%)	0.171
Weekly	20 (45.5%)	24 (54.5%)	
On occasions	81 (36.2%)	143 (63.8%)	
During work	27 (51.9%)	25 (48.1%)	
During your daily use, how many hours do you use lenses?			
Less than 8 hours	74 (34.1%)	143 (65.9%)	0.003
From 8 to 12 hours	73 (51.4%)	69 (48.6%)	
More than 12 hours	10 (31.3%)	22 (68.8%)	
From where do you get contact lenses?			
Hospital	8 (22.2%)	28 (77.8%)	0.1
Eyewear stores	74 (40.9%)	107 (59.1%)	
Beauty centers	50 (43.9%)	64 (56.1%)	
Internet websites	14 (35%)	26 (65%)	
Others	11 (55%)	9 (45%)	
Have you suffered eye dryness when using contact lenses?			
Yes	104 (44.3%)	131 (55.7%)	0.042
No	53 (34%)	103 (66%)	
Have you suffered eye redness when using contact lenses?			
Yes	110 (37.8%)	181 (62.2%)	0.105
No	47 (47%)	53 (53%)	

CL-related consequences

Table 6 represents the different CL-related consequences. Eye redness was the most common related consequence, observed in 291 (74.4%), followed by eye dryness in 235 (60.1%), and temporary vision loss in 63 (16.1%). Keratitis was less reported, occurring in 38 (9.7%). Almost three-quarters of the respondents, 279 (71.4%), reported that when they suffered from these consequences, they removed the lens, 99 (25.3%) went to the doctor, and 13 (3.3%) kept wearing them.

**Table 6:** Contact lens-related complications among the study participants (n=391), Jazan, Saudi Arabia. The data has been represented as Number (N) and Percentage (%).

Variable		N (%)
Have you been diagnosed with keratitis when using contact lenses?	Yes	38 (9.7%)
	No	353 (90.3%)
Have you suffered eye dryness when using contact lenses?	Yes	235 (60.1%)
	No	156 (39.9%)
Have you suffered eye redness when you use contact lenses?	Yes	291 (74.4%)
	No	100 (25.6%)
Have you suffered temporary vision loss while using contact lenses?	Yes	63 (16.1%)
	No	328 (83.9%)
What do you do when you suffer from these complications?	Remove the lens	279 (71.4%)
	Go to the doctor	99 (25.3%)
	Keep wearing them	13 (3.3%)

## Discussion

This population-based cross-sectional survey evaluates the level of knowledge and practice of contact lens (CL) wearing among adults in the Jazan region, Saudi Arabia. Over half of the study population were CL wearers, with three-quarters being female and two-thirds from urban areas. Females exhibited higher levels of knowledge and better practices toward CL wearing than males.

The overall prevalence of CL wearing in this study was even higher than the reported prevalence in two previous studies conducted in Jeddah and Mecca among college students [13-15]. This variation could be explained by differences in sample size and study population characteristics. The increased prevalence underscores the importance of high-level practices to enhance safe usage and decrease the risk of eye infections or inflammation.

Consistent with other studies, two-thirds or more of CL wearers are female [12, 16, 17]. This study found that three-quarters of wearers were female, possibly due to the fashion practices and cosmetic advantages offered by CLs that attract more females. Furthermore, there was a significant variation in knowledge levels related to gender, with females exhibiting excellent knowledge compared to males. A study in Al Ahsa, Saudi Arabia, among medical students of King Faisal University, involving 208 participants, found that female participants had higher total mean knowledge scores than males [10].

Previous studies have shown varied reasons for CL usage [9, 13, 18]. In this study, only 23.8% of the participants used them for therapeutic purposes, whereas 50.6% indicated cosmetic reasons. According to the observation, various earlier studies showed that CL wearers were more likely to wear them for cosmetic reasons [19], contrary to the observation made by Mahadevan et al. that CL wearers primarily wore them for therapeutic purposes.

Sleeping in CLs, a common risk behavior with a high relative risk for eye-related infections, was low in this study [16, 20, 21]. About 75.5% of participants were aware of the risks associated with sleeping while wearing CLs. This finding differs from those reported in Thailand and South Africa, where a slightly higher percentage of respondents slept with their lenses in [22, 23].

The risk of eye-related infection significantly increases when CLs are used after their expiration date [12]. In this study, more than half of CL wearers agreed to replace their CLs after their expiry date. Contrary to this good practice, Alasiri RA et al. [24] in Saudi Arabia reported that more than half of CL wearers did not replace lenses based on convenience.

The results of this study also showed that most of those who wear CLs replace the lens preserving liquid after each use. Contrary to this good hygiene practice, Khoza N et al. [23] and Ndedi SB [25] reported unsatisfactory CL case hygiene practices, and the respondents did not replace the preserving liquid in their cases regularly. This is one of the poor CL risk behaviors and has been identified as one of the leading risk factors for microbial keratitis in CL wearers [26]. Only 1.8% of the participants in the current study never cleaned their CL cases, unlike in the Cardona G et al. study [27], which reported that 19.1% of the participants never cleaned their CL cases. The majority of participants (69.3%) in the study population washed their hands before using CLs. Zhu Q et al. [5], Abahussin M et al. [17], and Vianya-Estopa M et al. [28] reported similar findings. To maintain good lens optics and ocular health, wearers must exhibit satisfactory CL hand hygiene practices.

It is generally recommended to consult ophthalmologists before using CLs because they can properly assess the eye health and advise on the correct lens size. They can also provide instructions on proper care and hygiene practice to prevent complications. Only one-third of the participants in this research agreed to wear CLs after consulting an ophthalmologist. Alasiri RA et al. [24] in Saudi Arabia, furthermore, reported in their study that two-thirds of the participants got their CLs from an optician's without first receiving an eye examination in the eye clinics. This inadequate approach may restrict hygiene education and increase the incidence of CL-related infections.

Never or rarely sharing CLs with others is a good practice observed in many previous studies [6, 7, 17]. In keeping with this, most of the participants in the current study never shared their CLs with others, which plays a part in decreasing the risk of CL-related infections

CL wearing may result in corneal and conjunctival complications secondary to hypersensitivity reactions, hypoxic changes, mechanical trauma, and infection [29]. In accordance with the literature, the most common CL-related adverse effects reported in this study were eye redness and dryness [11].

The results of this study have implications for educational initiatives and public health interventions. Increasing wearers' awareness and understanding of contact lens cleanliness and associated difficulties is essential, especially for male wearers. By addressing the gender gap in knowledge, healthcare workers can customize educational programs to reach specific demographics. Furthermore, lowering the risk of issues related to contact lens use can be accomplished by emphasizing the value of routine eye exams and good cleaning procedures.

Future research should seek to address these limitations and further explore the relationships between the variables studied. To expand on the findings of this study, more extensive research should be conducted on a larger scale, encompassing various regions and populations in Saudi Arabia. Longitudinal studies can shed light on how knowledge and practices shift over time. Qualitative research methods, such as interviews or focus groups, can also be used to gain a better understanding of the factors influencing contact lens use and barriers to adherence. These studies can help to inform the development of targeted interventions to improve contact lens hygiene and reduce complications.

This study has several areas of strengths and weaknesses. The main strength of this study is related to its community-based nature, which represents the level of knowledge and practice of CL wearing among adults in the Jazan region of Saudi Arabia. However, the study also has several limitations. The use of an online setting to collect the data may subject the study to selection bias by not enabling illiterate individuals to participate. Nonetheless, it is possible to argue that the majority of the community are literate and have appropriate access to internet services in a developing country such as Saudi Arabia, and thus, it is possible to argue that the study has good generalizability in the studied community.

## Conclusions

CL wearing is prevalent among the general population in the Jazan region. The overall knowledge of participants was excellent, and wearers exhibited adequate practice. This should be reinforced by having an eye examination before wearing CLs and by demonstrating proper wearing habits in terms of hygiene. This includes ceasing the sharing of CLs and using the CLs and preserving liquid only until the expiration date. Adherence to the recommended wearing schedule and expiration date is crucial in avoiding unwanted CL-related infections.
